# Human Brain Organoid Research and Applications: Where and How to Meet Legal Challenges?

**DOI:** 10.1007/s11673-024-10349-9

**Published:** 2024-07-05

**Authors:** M. Kataoka, T.-L. Lee, T. Sawai

**Affiliations:** 1 Graduate School of Humanities and Social Sciences, Hiroshima University, Higashi-Hiroshima, Japan; 2 Graduate Institute of Health and Biotechnology Law, Taipei Medical University, Taipei, Taiwan; 3 Institute for the Advanced Study of Human Biology, Kyoto University, Kyoto, Japan; 4 Centre for Biomedical Ethics, Yong Loo Lin School of Medicine, National University of Singapore, Singapore, Singapore

**Keywords:** Brain organoids, Consciousness, Personhood, Informed consent, Ownership, Transplantation

## Abstract

An ethical and legal framework is needed to regulate the rapidly developing human brain organoid research field properly. However, considering the legal issues involved in human brain organoid research remains underdeveloped and scattered. This article reviews the legal issues of human brain organoid research, grouping them into the following five broad themes: (1) consciousness, (2) legal status, (3) consent, (4) ownership, and (5) transplantation. The issues in each topic include both the urgent (e.g., appropriate forms of consent) and the speculative (e.g., protection of conscious human brain organoids). Therefore, we have attempted to be as explicit as possible about the timescale within which each issue will be realized and to prioritize each. Examining these issues has revealed legal issues specific to human brain organoid research and issues common to research in other fields. Further discussion of human brain organoid research from a legal perspective is needed in the future, considering discussions in related fields.

## Introduction

Research with three-dimensional neural tissue derived from human pluripotent stem cells, known as “human brain organoids,” has rapidly progressed. As this research is published, national and international bodies have addressed the ethical issues (International Society for Stem Cell Research 2021; Hyun et al. 2021; National Academies of Sciences, Engineering, and Medicine 2021). Adequate regulation requires an ethical and legal framework. However, legal perspectives on research in human brain organoids remain immature and scattered.

The legal issues related to human brain organoid research share concerns with all other research on human subjects, such as protecting the cell donor’s privacy. However, some issues are specific to brain organoid research. These can be organized into three broad categories (Taupitz 2022). First, issues regarding cell acquisition; second, issues regarding human brain organoids themselves; and third, issues related to their use. The first entails consent from cell donors. This is the most urgent issue and has thus been relatively well-examined (Boers and Bredenoord 2018; Greely 2020; Deuring 2022). Human brain organoid research has potential sensitivities for donors; accordingly, obtaining consent from the cell donor in an appropriate form is necessary. The second category involves issues regarding the treatment of possibly conscious human brain organoids and the legal status of human brain organoids. The former overlaps significantly with ethical problems, but the latter has not been examined much, despite its importance. Currently, the legal status of human brain organoids is a property (Lavazza and Pizzetti 2020; Taupitz 2022). However, recent research has pointed out the possibility of legal personhood (Jowitt 2023; Kataoka et al. 2023b). The third issue is manifold, depending on the potential uses of human brain organoids. Of particular importance, based on past discussions in stem cell research, are those related to the ownership of human brain organoids (Bredenoord et al. 2017) and those pertaining to their transplantation into animals or humans. Moreover, the very recent development of a field termed “Synthetic Biological Intelligence” (SBI), which can integrate human brain organoids as a part of computers (Kagan et al. 2022; Smirnova et al. 2023), further complicates various legal issues.

These legal issues are currently considered separately, but organizing them into a single view that serves as a reference point for further discussion would be helpful. Therefore, this study provides an overview of the legal issues in human brain organoid research and application (Table 1). For each topic, we sought to distinguish between current and future issues. For future issues, we clarify how likely they are to occur in the near future. We propose the issues that should be prioritized and encourage further discussions.

**Table 1:** An overview of the legal issues in human brain organoid research and application

Topics	Issues	Possible Measures
Consciousness	Uncertainty about consciousness of HBO	Precautionary measures, invention of method to detect consciousness and its standardization
	HBO with non-valenced experience	Protection of welfare
	HBO with sentience	Protection of welfare, harmonization with the treatment of animals
	HBO with advanced cognitive capacities	Protection of welfare, harmonization with the treatment of animals and humans
Legal Status	Synthetic Biological Intelligence	Recognition of legal personhood
	Citizens’ respect for human brain organoids	Recognition of legal personhood
	HBO with consciousness	Recognition of legal personhood
	HBO with the capacity to integrate a body	Recognition of legal personhood as natural persons
Consent	Sensitivities for donors	Adequate informed consent
	Uncertainty about consciousness of HBO	Adequate informed consent with precautionary protection of welfare of HBO
	HBO with consciousness	Adequate informed consent with protection of welfare of HBO
Ownership	Donors feeling personal relationship with HBO	Clarification of ownership
	Commercialization	Appropriate distribution of rights among stakeholders
Transplantation	Host animal’s welfare undermined	Protection of welfare of animals
	Host animal's capacities enhanced	Protection of welfare of animals
	Human clinical trial	Protection of the human subject
	Transplanted HBO becoming conscious	Protection of welfare of HBO

HBO means human brain organoids. Issues are listed in order of likely future scenarios

### 1. Consciousness

One of the most debated ethical concerns regarding brain organoids is the possibility that they will become conscious (de Jongh et al. 2022). Currently, many researchers believe that human brain organoids will not become conscious in the near future (International Society for Stem Cell Research 2021). However, several consciousness theories suggest that even existing human brain organoids could be conscious (Niikawa et al. 2022). Further, the feasibility depends on the definition of “consciousness.” For the sake of argument, we assume that human brain organoids can be conscious in principle and examine the legal implications of three types of “consciousness” in the order in which they could be easiest to realize. The first is a *non-valenced experience*—a mere sensory experience without positive or negative evaluations. The second is a *valenced experience* or *sentience—*an experience with evaluations such as pain and pleasure. The third is a more *developed cognitive capacity*. We assume that if any consciousness makes an entity a subject of (more complex) welfare, it may need to be legally (further) protected.

As a primitive form of consciousness, a non-valenced experience will, if possible, be realized earlier by human brain organoids than other forms of consciousness. However, the legal implications remain unclear. Suppose welfare consists solely of a good or bad experience. In that case, human brain organoids with a non-valenced experience have nothing to protect because they cannot have good or bad experiences. However, some argue that non-valenced experiences hold moral significance even without contributing to welfare. In addition, welfare may not be limited to experience as it has recently been adopted in animal ethics (Beauchamp and DeGrazia 2020). Adopting this perspective, even if human brain organoids possess only non-valenced experiences—or lack consciousness altogether—their basic sensory or motor capacities (Kataoka and Sawai 2023) or the possession of living or non-living bodies to utilize these capacities (Shepherd 2023), may warrant protection. This is contingent upon their potential contribution to the welfare of human brain organoids. Generally, the moral and legal examination of whether (and in what respects) entities with non-valenced experiences deserve protection is immature, at present. Given its feasibility in human brain organoids, this issue requires further exploration. Another important point is that once a human-derived tissue is conscious, regardless of how primitive it is, it may be a human individual. This point is addressed in Section 2.

In contrast to non-valenced experiences, there are well-established legal practices concerning sentient entities because we have laws and regulations to protect the welfare of animals. While they are often applied only to vertebrates, there has been much debate regarding extending their scope to invertebrates. In principle, animal welfare laws can be extended to human brain organoids. For, two general justifications for animal welfare laws—the protection of animal welfare and the protection of public feelings toward animals—can also be applied to human brain organoids (Lavazza and Pizzetti 2020). Human brain organoids will be sentient in the distant future. However, if this happens, ethical principles employed in treating laboratory animals should govern the handling of human brain organoids (Koplin and Savulescu 2019).

Finally, if human brain organoids become more cognitively advanced, they will require stricter protection. However, given that this will probably not happen until the distant future and that there are precedents for various animals, including humans, this issue is probably not a priority.

Although some methods have been proposed to detect consciousness in human brain organoids (Lavazza and Massimini 2018), an epistemological question about how to detect all three forms of consciousness remains. For now, scholars have proposed precautionary measures when scientific uncertainties exist to protect the welfare of brain organoids (Koplin and Savulescu 2019; Birch and Browning 2021; Niikawa et al. 2022). Relatedly, if some method of detecting consciousness is invented, it should be standardized internationally for regulatory purposes (Voeneky 2022).

### 2. Legal Status

Currently, the legal status granted to human brain organoids is a property—similar to all other human-derived materials. This situation is not likely to change soon, although they may be treated as legal persons in the future (Kataoka et al. 2023b).

Whether human brain organoids have legal personhood will be one of the most urgent issues when considering future information processing systems incorporating brain organoids—a type of SBI system (Smirnova et al. 2023). Research is underway in which cultivated human neural tissues on silicon chips play simple video games (Kagan et al. 2022). It seems a matter of time before these systems will generate text, images, or musical pieces. This raises the question of whether SBI systems should be granted legal personhood to protect intellectual property rights—as is currently being discussed for artificial intelligence systems. The next issue relates to welfare protection. Even for animals, welfare is not sufficiently protected because their legal personhood is not recognized. The same problem may arise in future conscious human brain organoids (Jowitt 2023). Third, cases in which legal personhood is assigned to natural entities, such as rivers, show that recognizing legal personhood can reflect social beliefs or values about objects. If a society believes that we have a special responsibility towards human brain organoids, this may lead to the granting of legal personhood.

Furthermore, it is important to consider the possibility of human brain organoids becoming natural persons, i.e., human individuals (Kataoka et al. 2023b). This is a possibility for the distant future but one that should be seriously considered because, if this were the case, some case of the production of human brain organoids would be tantamount to human reproductive cloning (Kataoka et al. 2023c). In many countries, brain death, or the irreversible loss of the brain’s capacity to integrate the body, is a criterion for the death of the human individual. This may imply that a sufficiently mature human brain organoid that can integrate the body could be a human individual. Even if the integrative capacity is too difficult for human brain organoids to achieve, the same problem may arise for the most primitive consciousness. In the bioethical literature on brain death, a human body with even minimal signs of consciousness is often considered a human individual (Moschella 2016; Huang and Bernat 2019). This can mean that conscious human brain organoids can be human individuals. Certainly, under current legal systems, human brain organoids, regardless of how mature they might be, cannot be natural persons just because they are not legally “born” (delivered from the mother’s body) (Lavazza and Pizzetti 2020; Taupitz 2022). However, given the recent development of technologies to culture various human tissues from scratch, this traditional understanding of birth may be a shortcoming of current legal systems. Notably, the traditional understanding of birth is also challenged—independent of organoid technology—by debates about abortion or the development of medical technology such as fetal surgery and artificial uteruses (Romanis 2020). Furthermore, the notion of legal personhood is profoundly influenced by cultural contexts, indicating that the categories of “born” individuals and natural persons have not always coincided (Stewart 2002). Therefore, the potential classification of human brain organoids as natural persons hinges on societal perceptions and the attributed value to these entities.

### 3. Consent

Since human brain organoids are produced from human pluripotent stem cells, appropriate consent must be obtained from the cell donors. While many concerns about consent are shared with other research projects, some concerns are unique to human brain organoid research.

The first and most urgent concern is related to blanket and broad consent mechanisms, which fail to delineate the scope of future research utilizing the donated cells. Generally, such consent processes are inappropriate for “controversial uses” of donated cells to which some donors may be expected to object (Greely 2020). Such research carries a high risk of violating the donors’ rights to self-determination. Research on human brain organoids can fall under this category. In an interview study with Dutch citizens, brain organoids were perceived to be of particular ethical concern because of their potential for consciousness (Haselager et al. 2020). Until more extensive social surveys are conducted to determine people’s attitudes towards brain organoid research and possible reasons for their opposition, (re)obtaining project-specific informed consent is more desirable (Greely 2020).

However, human brain organoid research may pose challenges to obtaining project-specific informed consent from cell donors. Even if it is safe to state that current immature human brain organoids are not conscious, a situation of high uncertainty regarding consciousness may arise in the future. This uncertainty may undermine the nature of informed consent, raising questions regarding its validity (Greely 2021; Mollaki 2021). Here, one could argue that accepting high uncertainty is a part of the donor’s right to self-determination (Deuring 2022). However, even if so, there is another challenge regarding consciousness (Kataoka et al. 2024). If conscious human brain organoids are welfare subjects, their welfare will be unjustly violated in research unless adequate consideration is taken. Donating cells for such research may exceed the appropriate scope of the donor’s right to self-determination because it involves harm to a third party, the human brain organoid. This indicates that protecting the welfare of human brain organoids may be a precondition for obtaining valid informed consent.

### 4. Ownership

Human brain organoids—as other biological materials collected for research purposes—are owned by researchers and research institutions, not by cell donors. However, human brain organoids pose several pressing challenges to current legal practice.

First, cell donors may feel differently about brain organoids than donated cells or other products. Specifically, donors may feel a strong personal connection to the human brain organoids. Alternatively, the donor may feel that the human brain organoids belong to them. Existing empirical studies have yielded ambivalent results (Haselager et al. 2020; Bollinger et al. 2021). Some people feel stronger personal connections to brain organoids than other organoids, although this does not necessarily mean people claim ownership. However, it is safe to say that if the donor feels that the brain organoids are their own and if this feeling is not adequately considered—at least in the consent process for cell donation—this may lead to ownership disputes between the donor and researchers. Further research is needed to clarify donor attitudes.

A related problem may arise from the monetary value of brain organoids (Bredenoord et al. 2017). Brain organoids are useful in pharmaceutical research, and their monetary value is expected to increase. The advancement of SBI technology has the potential to substantially increase the economic valuation of human brain organoids. When researchers perform innovative interventions, such as genetic editing of brain organoids, they can have intellectual property rights that permit them to patent such interventions or their results. Patented tissues derived from human embryonic stem cells are not permitted in the EU, nor are brain organoids derived from them as well (Hostiuc et al. 2019; Wolff 2024). This could lead to an unfair distribution of the benefits among jurisdictions. While brain organoids derived from human induced pluripotent stem cells are patentable in the EU, the patentability of future conscious brain organoids may be precluded (Wolff 2024). Further, while the researcher can obtain the financial benefit generated from the brain organoids, the donor, who does not have ownership, cannot. This may be unfair to the donor and points to the need to share the benefits gained from medical and non-medical applications of human brain organoids with the donor (Boers et al. 2016; Savulescu et al. 2022). However, it also makes sense to prevent the commercialization of the human body by not providing the donor with any financial benefits. To address this dilemma, we must consider appropriately regulating and distributing relevant rights, including ownership among stakeholders (Boers et al. 2016; Bredenoord et al. 2017; Kagan et al. 2023).

### 5. Transplantation

Human brain organoids have been transplanted into the brains of animals such as mice and rhesus monkeys (Mansour et al. 2018; Daviaud et al. 2018; Kitahara et al. 2020; Revah et al. 2022). Transplanted brain organoids become more mature than *in vitro*. Animal transplantation is a preliminary step to human transplantation—one of the expected medical applications of human brain organoids (Chen et al. 2019b). Both animal and human transplants require adequate regulation.

Regarding animal transplants, the most pressing issue is the welfare of host animals. Human brain organoid transplantation is clearly invasive, and decreased brain function has been reported (Mansour et al. 2018). Therefore, this type of research must be conducted with appropriate research objectives and due consideration for the well-being of the animals (Hoppe et al. 2022). However, ethicists have recently pointed out that animal transplants of human brain organoids may not have been performed ethically and that the behaviour of animals has not been fully evaluated, making ethical assessments difficult (Barnhart and Dierickx 2023). This may suggest that animal transplantation of human brain organoids should be regulated more strictly, and more rigorous evaluations of the purpose of the research and the physiology, behaviour, and welfare of animals are needed than currently conducted.

No health problems were reported in host rats in one of the most recent animal transplants (Revah et al. 2022; Hyun 2022). If the host animal can regain normal brain function after the transplantation, the next issue is enhancing the functions by transplanted human brain organoids. Some enhanced functions may require further protection to ensure animal welfare, particularly in instances where animals are subjected to stress (Bassil and Horstkötter 2023). Thus, it is necessary to identify enhancements and fully evaluate them in a sufficiently individualized manner (Chen et al. 2019a). Indeed, Dong et al. (2021) reported an elevated fear response in mice implanted with human brain organoids, highlighting the imperative for heightened attention to the welfare of these animals.

In addition, issues related to the consciousness and welfare of transplanted human brain organoids are often overlooked (Kataoka et al. 2023d). While issues related to consciousness will generally be addressed in the future, this point is likely to become a problem relatively soon. Since human brain organoids mature more *in vivo* than *in vitro*, consciousness will likely be realized first in animal transplants. Thus, issues surrounding consciousness and welfare protection should first be considered for human brain organoids *in vivo* rather than *in vitro*. Animal transplants must be carefully regulated if (advanced) conscious brain organoids are not to be produced (even unintentionally).

Since transplantation in humans is likely to be feasible only in the more distant future, we can only make some basic claims. Clinical trials are highly invasive, requiring a strong reduction in relevant risks and appropriate informed consent. The possibility of psychological alterations due to transplanted organoids would be particularly concerning among the various related risks (Sawai et al. 2022). However, concerns about personality transformation may be over-inflated for technologies such as deep brain stimulation (Gilbert et al. 2021). Thus a more thorough evaluation of the physiology and behaviour in animal transplantation may be needed from this perspective as well.

## Conclusion

Ethical considerations should guide and inform how legal responses in regulating novel research; however, it is often difficult to formulate appropriate laws. We reviewed five topics of legal issues specific to brain organoid research that current and future laws should address. They include imminent and hypothetical issues, and we try to clarify the timescale in which each case might be actualized. Such time ordering has another meaning besides identifying the issues that need to be urgently addressed. Currently, various concerns regarding human brain organoids have been exaggerated (Presley et al. 2022; Kataoka et al. 2023a). When addressing normative issues around cutting-edge research or technologies like brain organoids research, it is important to be explicit about how pressing the issue is to avoid raising unnecessary fears. We hope the nuanced overview provided here will encourage more scholarly discussions on the legal issues of human brain organoid research while avoiding the exaggeration of possible problems.

Finally, such discussions should lead to the harmonization of regulatory frameworks. As we have shown, the legal issues of human brain organoid research significantly overlap with the legal issues of other stem cell and animal research, abortion, cutting-edge medical technologies, and artificial intelligence. For the regulation of one research area to be reasonable, it must be in harmony with the regulation of another. Furthermore, in a globalized world, novel or disruptive research’s social and ethical implications are no longer confined to a particular jurisdiction; addressing these common socio-ethical concerns requires a coherent global response. Unfortunately, there is currently no international legal framework for regulating human brain organoid research (Voeneky 2022). This might create regulatory loopholes. Scientists or practitioners may prefer to work in a relatively lax jurisdiction that regulates research and applications, creating leeway that may contravene shared ethical standards. Given the trends in stem cell research to date, such an imbalance could lead to an increase in ethically questionable research and, in the future, social problems, such as medical tourism. Articulating a legal or regulatory framework—reflecting a shared set of ethical principles to guide research—will embed the ethics vocabulary as science advances.
